# An Optimal Control Strategy for DC Bus Voltage Regulation in Photovoltaic System with Battery Energy Storage

**DOI:** 10.1155/2014/271087

**Published:** 2014-04-24

**Authors:** Muhamad Zalani Daud, Azah Mohamed, M. A. Hannan

**Affiliations:** ^1^School of Ocean Engineering, Universiti Malaysia Terengganu, 21030 Kuala Terengganu, Malaysia; ^2^Department of Electrical, Electronic & Systems Engineering, Universiti Kebangsaan Malaysia, 43600 Bangi, Selangor, Malaysia

## Abstract

This paper presents an evaluation of an optimal DC bus voltage regulation strategy for grid-connected photovoltaic (PV) system with battery energy storage (BES). The BES is connected to the PV system DC bus using a DC/DC buck-boost converter. The converter facilitates the BES power charge/discharge to compensate for the DC bus voltage deviation during severe disturbance conditions. In this way, the regulation of DC bus voltage of the PV/BES system can be enhanced as compared to the conventional regulation that is solely based on the voltage-sourced converter (VSC). For the grid side VSC (G-VSC), two control methods, namely, the voltage-mode and current-mode controls, are applied. For control parameter optimization, the simplex optimization technique is applied for the G-VSC voltage- and current-mode controls, including the BES DC/DC buck-boost converter controllers. A new set of optimized parameters are obtained for each of the power converters for comparison purposes. The PSCAD/EMTDC-based simulation case studies are presented to evaluate the performance of the proposed optimized control scheme in comparison to the conventional methods.

## 1. Introduction

Despite the many advantages offered by photovoltaic- (PV-) based renewable energy (RE) generation, it suffers from unpredictable environmental conditions and abrupt changes in system loads. In addition, when the PV-based RE generation is grid connected, the possibility of utility grid fault at the point of common connection (PCC) might result in a system breakdown or the interruption of power supplied to critical loads. One of the typical challenges in integrating such a variable generation to utility grid is in controlling the DC bus voltage stability within the power conversion system [1, 2]. The disturbances, such as varying environmental conditions, system loads, and fault occurrences, cause the DC bus voltage to fluctuate, overshoot or undershoot, and sag or dip [3]. Poor regulation of the DC bus voltage may result in system instability and inferior efficiency of PV systems. The problems above can be attributed to the poor dynamics of PV control systems in comparison to the transient time from the disturbances [4].

The instability of DC bus voltage may propagate over the PV system network, where, in some cases, the requirement for fast dynamic compensation devices, such as diesel generators or the battery energy storage (BES) for power fluctuation management and fault ride by mitigation, is indispensable. Lead-acid-type battery is currently the most preferred storage method for most RE based integration issues because their mature technology provides a reasonable trade-off between cost and performance [5, 6]. The importance of DC bus voltage regulation based on BES is that it provides a constant DC bus voltage seen by the grid side voltage-sourced converter (G-VSC), resulting in efficient power conversion while protecting the DC bus capacitor and the G-VSC valves against overvoltage stress.

Technically, the variation in DC bus voltage can be mitigated by adjusting the duty cycle of the power converters responsible for voltage regulation, which is normally performed by the proportional integral (PI) compensator [7–10]. The PI controller is commonly characterized by its distinct parameters, namely, the proportional gain, *k*_*p*_, and the integral gain, *k*_*i*_, respectively. From the literature, several conventional tuning strategies that have been in use include the hand tuning or trial and error, Smith, Ziegler-Nichols, and pole placement methods [7]. However, these tuning strategies require the determination of system transfer function, which makes the method more complex [8]. A number of optimization methods have been developed for fine-tuning converter control parameters [9, 10], but only few applications used the simplex algorithm. From previous studies, applications of the simplex algorithm are in controlling FACTS devices [11, 12] and vehicular control systems that employ bidirectional converters [13, 14]. This paper addresses the issue of improving DC bus voltage regulation by using G-VSC and BES with a bidirectional DC/DC buck-boost converter for the efficient performance of grid-connected PV systems. The BES connected to the DC bus of the PV system can enhance the system's dynamic performance by controlling the charging/discharging of BES with a bidirectional converter when the system is subjected to varying disturbances. The optimal controls of the G-VSC and the bidirectional DC/DC converter are investigated in the proposed control methodologies. First, the control methods of the G-VSC, namely, the voltage-mode and current-mode controls, are assessed. Second, the control parameters of the G-VSC and the bidirectional DC/DC are optimized using the simplex optimization algorithm to obtain the optimum set of parameters for the control systems.  Section 2 briefly describes the system configuration and operation principle of the considered grid-connected PV with BES.  Section 3 provides the modeling details of the PV and BES.  Section 4 describes the proposed control strategies for the G-VSC and the bidirectional DC/DC converter.  Section 5 discusses the DC bus regulation strategy and the control parameter optimization in detail. Sections 6 and 7 present the simulated results and some concluding remarks, respectively.

## 2. System Configuration and Operation

**Figure 1:**
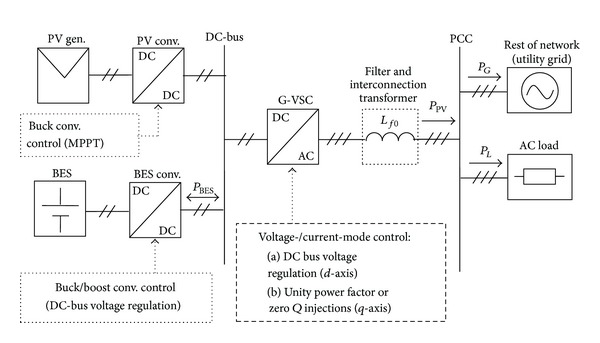
System configuration/control of the grid-connected PV with BES.

The configuration of the considered single-stage grid-connected hybrid PV/BES system is as shown in Figure 1. The system consists of a PV array (PV gen.) interfaced to the DC bus through a buck DC/DC converter with a maximum power point tracking (MPPT) controller. The G-VSC facilitates the MPPT operation through regulation of the DC bus voltage as well as transfer of power from the DC bus to the utility grid. In addition, the G-VSC provides synchronization of the PV system with the grid during startup or reconnection after system islanding.

As can be seen from Figure 1, to enhance the DC bus voltage regulation, BES is used where it is interfaced via a bidirectional buck-boost converter (BES conv.) which controls the charge/discharge processes during severe operating conditions such as abrupt change in solar irradiation level and fault occurrences. From the G-VSC AC output terminals, the hybrid subsystem is connected to the utility grid at the PCC through a low-pass filter and an interconnection transformer that is represented by an inductor. These components are responsible for filtering harmonics and isolating the entire system from the utility grid. The transformer steps up the voltage level of the PV system from 0.23 kV to 11 kV line-to-line RMS voltage. The PV/BES system injects total power, *P*_*G*_, to the utility grid in which in this case the utility grid network is based on a standard medium voltage distribution system [15].

## 3. System Modeling

This section provides the detailed models of the system components which are simulated using the PSCAD/EMTDC transient simulator [16].

### 3.1. Modeling of PV Array

**Figure 2:**
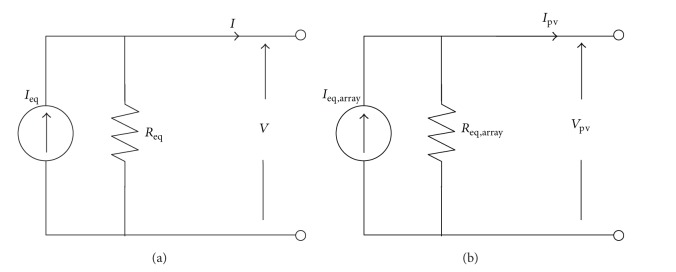
Norton equivalent circuit of the (a) PV module and (b) PV array.

The inclusion of the PV model in PSCAD/EMTDC is based on the Norton equivalent circuit given in Figure 2 [17]. Assuming that the PV modules are of the same type and are subjected to similar environmental conditions, a PV array model (Figure 2(b)) can be represented by a combination of series-connected, *N*_*s*_, and parallel-connected, *N*_*p*_, modules. The current and resistance of such PV array model are given by

(1) $$\begin{array}{c} I_{\text{eq},\text{array}}=N_{p}I_{\text{eq}}=N_{p}I_{i}\frac{R_{p}}{R_{s}+R_{p}},\\ R_{\text{eq},\text{array}}=\frac{N_{s}}{N_{p}}R_{\text{eq}}=\frac{N_{s}}{N_{p}}\left(R_{s}+R_{p}\right), \end{array}$$

where *R*_*s*_ and *R*_*p*_ are the series and parallel resistance of an ideal single diode PV cell model, representing the structural resistance and leakage current of the *p*-*n* junction, respectively [18]. *I*_*i*_ is the terminal current of an ideal PV module derived from the ideal PV cell model, which has the following form [17]:

(2) $$\begin{array}{c} I_{i}=I_{g}\left[I_{0}\left\{\exp\left(\frac{\beta }{\alpha }\cdot \frac{R_{p}}{R_{s}+R_{p}}\left(V+R_{s}I\right)\right)-1\right\}\right], \end{array}$$

where *I*_*g*_ is the cell's photocurrent, *I*_0_ is the dark current, and *α* is the diode ideality factor. *β* is the inverse thermal voltage defined as *β*(*T*) = *q*/*kT*, where *q* is the electron charge, *k* is Boltzmann's constant, and *T* is the *p*-*n* junction temperature. Except for *α* (assumed in this study as 1.5), all of the circuit parameters given in (2) are functions of the PV device type obtained from the manufacturer datasheet [17, 19].

### 3.2. Modeling of Lead-Acid Battery

Many options are available for the selection of a suitable battery model for a range of applications. These options include the simple voltage source model, the Thevenin model [20], generic models [21, 22], and dynamic and more realistic models [23, 24] that consider the nonlinear characteristics of battery parameters. However, to avoid excessive complexity with the consideration of the dynamic behavior of the battery cell, this study considered a generic model described in [21]. The model for the lead-acid battery is constructed based on two important parameters, namely, the terminal voltage, *V*_bat_, and state of charge, SOC, which represent the behavior of the battery. *V*_bat_ and SOC can be calculated as functions of battery current, *I*_bat_, as follows:

(3) $$\begin{array}{c} V_{\text{bat}}=E_{\text{bat}}-R_{\mathrm{int}}I_{\text{bat}},\\ \text{SOC}=100\left(1-\frac{\int I_{\text{bat}}dt}{Q_{\text{bat}}}\right), \end{array}$$

where *E*_bat_ is the open circuit voltage of the battery that is modeled using a controlled voltage source calculated as follows:

(4) $$\begin{array}{c} E_{\text{bat}}=E_{0}-K\frac{1-\text{SOC}}{\text{SOC}}Q+A\exp\left\{-B\left(1-\text{SOC}\right)Q_{\text{bat}}\right\}. \end{array}$$

In (3)–(4), *R*_int⁡_ and *Q*_bat_ are the battery internal resistance and capacity, respectively. *E*_0_ represents the battery open circuit voltage that lies between the fully charged voltage and the exponential voltage of the battery discharge curve, *K* is the polarization voltage, *A* is the exponential voltage, and *B* is the exponential capacity. From (4), the model accounts for both the normal voltage part and the exponential part represented by the second and third terms, respectively. Following the parameter extraction procedure provided in [21], all model parameters can be extracted from the manufacturer discharge curves, which are available in the battery datasheet [25].

## 4. Control of Power Converters

As shown in Figure 1, the power converters are comprised of the DC/DC buck converter, G-VSC, and BES DC/DC buck-boost converter, respectively. Since G-VSC and BES DC/DC buck-boost controls directly influence the voltage regulation of PV system DC bus, the control schemes are described in detail in the following subsections.

### 4.1. G-VSC Control

In general, the aim of the G-VSC control scheme is to control the active and reactive power transfer between the PV system and the utility grid. The active current component (*d*-axis) of the G-VSC is used to regulate the DC bus voltage of the PV system, whereas the reactive current component (*q*-axis) is used to ensure that the system injects reactive power at approximately unity power factor. The general operation of the PV system has to follow the regulations stated in the IEEE 1547 and UL1741, which states that the PV inverter is only allowed to inject power at unity power factor [26]. The G-VSC control developed is based on two different methods, namely, the voltage-mode [1] and current-mode [17] controllers, respectively.

#### 4.1.1. Voltage-Mode Control

**Figure 3:**
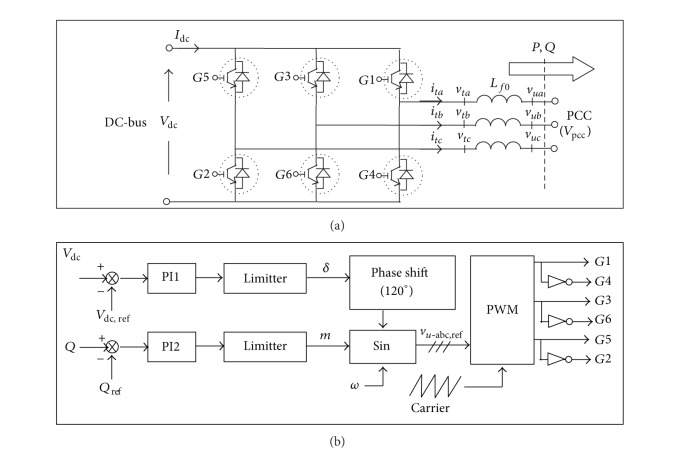
G-VSC control scheme, (a) G-VSC circuit, and (b) voltage-mode controller.

Figure 3 shows the detailed G-VSC circuit with the control block diagram based on the voltage-mode control scheme. The controller has merits, such as reduced control complexity and low number of control loops [1]. Since it is an open-loop control scheme, the measures taken to protect the G-VSC valves against instantaneous over current or network faults are by limiting the output references from PI controllers using the saturation blocks (limiter) shown in Figure 3(b). The principle for the active, *P*, and reactive, *Q*, power controls via G-VSC is expressed as follows:

(5) $$\begin{array}{c} P=\frac{V_{\text{pcc}}V_{\text{dc}}}{X_{s}}m\sin\left(\delta \right),\\ Q=\frac{V_{\text{pcc}}}{X_{s}}\left[V_{\text{pcc}}-mV_{\text{dc}}\cos\left(\delta \right)\right], \end{array}$$

where *V*_pcc_ is the output fundamental component voltage at the PCC, *V*_dc_ is the DC bus voltage, *X*_*s*_ = *ωL*_*f*0_ is the sum of inductances for filter inductance and coupling transformer inductance, *δ* is the angle, and *m* is the modulation index. Notably, based on the PWM principle, the relationship between the output fundamental component voltage at the G-VSC terminal, *V*_pwm_, and the DC bus voltage can be expressed as *V*_pwm_ = *mV*_dc_. *P* and *Q* are proportional to *δ* and *m*, respectively, when *L*_*f*0_ is optimally designed and *δ* is less than 10° [27]. This control strategy results in a simple voltage-mode control system design where active power is controlled by the value of *δ* and reactive power is regulated using *m* through PI1 and PI2, respectively.

As shown in Figure 3(b), PI1 generates a reference angle, *δ*, for the PWM by processing the error of the DC bus voltage to ensure that the flow of power is constantly directed from the PV to the grid while regulating the DC bus voltage. Consequently, PI2 generates the output reference, *m*, that processes the error of reactive power from its reference value and keeps the net power injected to the grid at the unity power factor (*Q*_ref_ = 0). Importantly, the value of *m* is controlled to make it remain less than unity (i.e., the linear modulation region), with the optimal value preferably close to unity, to ensure a reasonable efficient power conversion by the G-VSC. The output terminal voltage of G-VSC is regulated at *ω* which is the reference fundamental frequency at the PCC.

#### 4.1.2. Current-Mode Control

**Figure 4:**
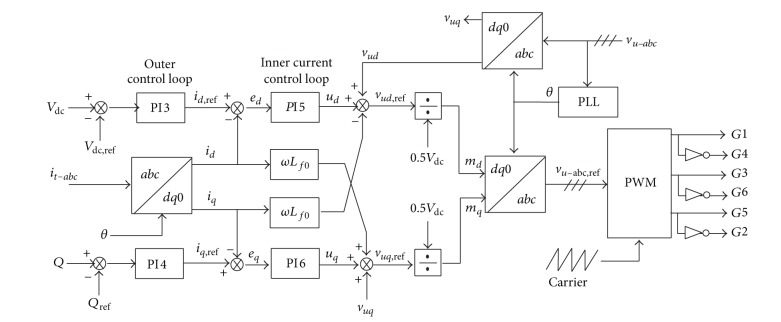
G-VSC control based on the current-mode scheme.

The current-mode control is a closed-loop current control method, in which the phase angle and magnitude of the VSC terminal voltage are controlled in a *d*-*q* rotating reference frame. This control approach imposes direct regulation on the G-VSC current, which avoids the vulnerability of G-VSC valves when they are subjected to large transient currents, such as during network faults and abrupt load changes. Referring to the G-VSC circuit shown in Figure 3(a), the control block diagram for the current-mode control scheme is as shown in Figure 4.

As can be seen in Figure 4, a phase-locked loop (PLL) is utilized to extract the angle, *θ*, of the grid voltage, that is to be used by the* abc*-to-*dq0* or* dq0*-to-*abc *transformation blocks. In the PLL, the PCC voltage vector (*v*_*u*-*abc*_) is projected on the *d* and *q* axes of the *d*-*q* frame and the *d*-*q* frame is rotated in such a way that *v*_*uq*_ is forced to zero [17]. Settling *v*_*uq*_ at zero makes the *d*-axis of the *d*-*q* frame aligned with the grid voltage vector, and the *d*-*q* frame rotational speed becomes equal to the grid angular frequency [1]. Regarding this, the synchronization scheme based on PLL makes the value of *P* and *Q* proportional to and can be controlled by *i*_*d*_ and *i*_*q*_, respectively, as follows:

(6) $$\begin{array}{c} P=\frac{3}{2}v_{ud}i_{d},\\ Q=-\frac{3}{2}v_{ud}i_{q}. \end{array}$$

In this case, *P* is represented as a DC bus voltage (*V*_dc_), in which the processing of the *V*_dc_ and *Q* errors is based on the PI compensators (PI3 and PI4) to generate the reference currents, *i*_*d*,ref_  and *i*_*q*,ref_, respectively, which can be used by the closed-loop current controller. *i*_*d*,ref_  and *i*_*q*,ref_  can be expressed as follows:

(7) $$\begin{array}{c} i_{d,\text{ref}}=\left(k_{pd3}+k_{id3}\frac{1}{s}\right)\left(V_{\text{dc}}-V_{\text{dc},\text{ref}}\right),\\ i_{q,\text{ref}}=\left(k_{pq4}+k_{iq4}\frac{1}{s}\right)\left(Q-Q_{\text{ref}}\right), \end{array}$$

where *k*_*p*_ and *k*_*i*_ are the proportional and integral gains, respectively, used in the PI3 and PI4 corresponding to *d*- and *q*-axes components, respectively.

The current-mode control scheme within the inner current loop control of Figure 4 independently controls *i*_*d*_ and *i*_*q*_ to make them rapidly track their respective current commands (*i*_*d*,ref_ and *i*_*q*,ref_), which can be represented in *d*-*q*-frame equivalents as follows:

(8) $$\begin{array}{c} L_{f0}\frac{di_{d}}{dt}=\omega L_{f0}i_{q}+0.5V_{\text{dc}}m_{d}-v_{ud},\\ L_{f0}\frac{di_{q}}{dt}=-\omega L_{f0}i_{d}+0.5V_{\text{dc}}m_{q}-v_{uq}, \end{array}$$

where *L*_*f*0_ is the equivalent filter inductance that represents the total inductance of the grid side filter and the transformer leakage reactance, *V*_dc_ is the DC bus voltage, and *ω* is the grid frequency. It should be noted that the terms 0.5*V*_dc_ *m*_*d*_ and 0.5*V*_dc_ *m*_*q*_ in (8) represent the G-VSC AC-side terminal voltages, *v*_*td*_ and *v*_*tq*_, respectively, in which *m*_*d*_ and *m*_*q*_ are the corresponding *d*-axis and *q*-axis control inputs (PWM modulation signals), while *v*_*ud*_ and *v*_*uq*_ are the exogenous inputs, respectively [17].

Rearranging (8) and considering *u*_*d*_ = *L*_*f*0_ *di*_*d*_/*dt* and *u*_*q*_ = *L*_*f*0_ *di*_*q*_/*dt*, 0.5*V*_dc_ *m*_*d*_ = *v*_*ud*,ref_, and 0.5*V*_dc_ *m*_*q*_ = *v*_*uq*,ref_ yield the following:

(9) $$\begin{array}{c} v_{ud,\text{ref}}=v_{ud}+u_{d}-\omega L_{f0}i_{q},\\ v_{uq,\text{ref}}=v_{uq}+u_{q}+\omega L_{f0}i_{d}, \end{array}$$

where *v*_*ud*,ref_  and *v*_*uq*,ref_  are the output voltage references from the current controller, *u*_*d*_ is the output of the compensator PI5 (*k*_*pd*5_ and *k*_*id*5_) that processes the error signal of *e*_*d*_ = *i*_*d*,ref_ − *i*_*d*_, and *u*_*q*_ is the output of compensator PI6 (*k*_*pq*6_ and *k*_*iq*6_) that processes the error signal of *e*_*q*_ = *i*_*q*,ref_ − *i*_*q*_. The generated reference modulation index from the current controllers, *m*_*d*_ and *m*_*q*_, is transformed back to the phase equivalents for use of the PWM generator using the* dq0*-to-*abc* transformation block.

### 4.2. BES DC/DC Buck-Boost Converter Control

**Figure 5:**
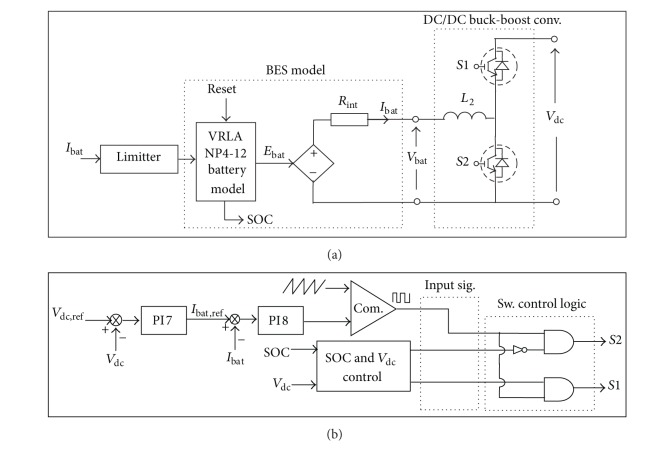
BES buck/boost converter control: (a) converter circuit and (b) control scheme.

Control of the charging/discharging of BES is achieved by using a buck-boost converter circuit with two PI controllers, as shown in Figure 5, where PI7 processes the DC bus voltage discrepancies during disturbances to make the bus voltage follow the voltage set point set as *V*_dc,ref_ = 500 V. The internal current control loop is also adopted for the battery current controller compensated by PI8. The output signal from PI8 is passed to the PWM generation circuit where the logic circuit is used for the decisions, including the charge, discharge, or halt modes of operation. The switch S1 is triggered and S2 is zero during the boost (discharge) mode and vice versa during the buck (charge) mode to absorb power from the DC bus. Both S1 and S2 become zero (halt mode) when no regulation signal is transferred.

**Figure 6:**
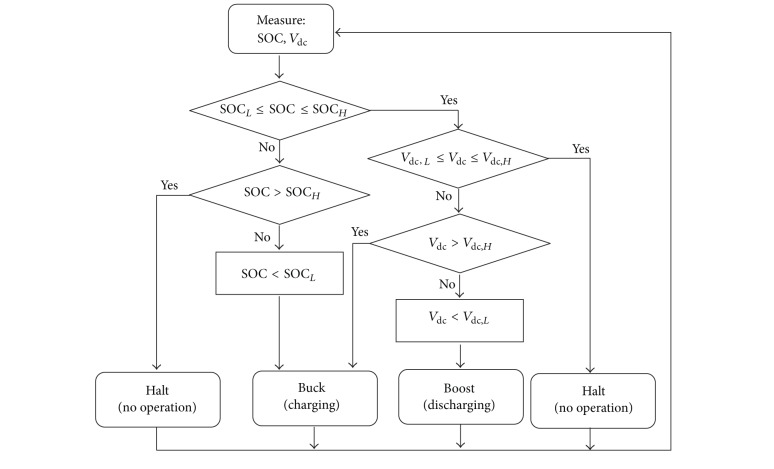
Flowchart for SOC and BES power charge/discharge controls.

Figure 6 shows the algorithm developed for SOC and bus voltage control to ensure a secure and optimal operation of BES. The upper and lower limits of SOC denoted as SOC_*H*_ and SOC_*L*_ are defined to avoid overcharge or deep discharge of BES. The upper and lower SOC levels avoid operation at exponential capacity region which may affect the magnitude of current drawn at the battery terminal. Preferably, for lead-acid battery, the recommended useable capacity should not exceed 70% of the total capacity especially when considering continuous charge discharge operation [25]. Consequently, the upper and lower boundaries of the DC bus voltage control are also considered to avoid unnecessary charge/discharge. In this case, the battery is set to charge/discharge only when the deviation of DC bus voltage exceeds a certain range denoted as *V*_dc,*H*_ and *V*_dc,*L*_, respectively. The algorithm utilizes the input references of the SOC and the DC bus voltage where the output logic from the algorithm provides input for the control logic circuit for determining the operating modes (see Figure 5).

## 5. Optimal DC Bus Voltage Regulation Strategy

In conventional grid connected PV system, without BES, the regulation of DC bus voltage is achieved solely through the G-VSC. However, with BES connected to the DC bus, enhanced control strategy can be achieved through regulation by both G-VSC and BES buck-boost converters. Regarding this, it is important to develop an optimal control scheme to boost the regulation performances of these power converters so as to minimize the stress received along the DC bus during severe disturbance condition. The effect of bus voltage fluctuation is related to power imbalance caused by fluctuations that originate from various sources of disturbances such as sudden change of solar irradiation, utility grid fault, and load step change in the vicinity. Such a power imbalance causes extra energy, Δ*E*, which is related to the DC bus capacitor, *C*_dc_, as follows:

(10) $$\begin{array}{c} \Delta E=\overset{}{\underset{T_{s}}{\int }}\Delta Pdt=\frac{1}{2}C_{\text{dc}}\left(V_{\text{dc}1}^{2}-V_{\text{dc}0}^{2}\right), \end{array}$$

where *V*_dc0_ and *V*_dc1_ represent the DC voltage at the start and end of the period *T*_*s*_, respectively, and *C*_dc_ is the total DC capacitance. In (10), *C*_dc_ largely influences the variation in DC bus voltage, and thus the response of the system compensators for DC bus voltage regulation must be sufficiently fast.

From Figure 5(b), the battery current reference, *I*_bat,ref_, for charge/discharge operation of buck-boost converter is provided by PI7 in which the DC bus voltage is regulated using the compensator as follows:

(11) $$\begin{array}{c} I_{\text{bat},\text{ref}}=\left(k_{pb7}+k_{ib7}\frac{1}{s}\right)\left(V_{\text{dc},\text{ref}}-V_{\text{dc}}\right), \end{array}$$

where *k*_*pb*7_ and *k*_*ib*7_ are the control parameters.

To improve voltage regulation so as to increase the dynamic performance of the system, the control parameters for the BES DC/DC converter and the G-VSC need to be optimized. The conventional hand-tuning method of the PI controllers used for the control system is improved by using the simplex optimization toolbox available in the PSCAD/EMTDC [28].

The simplex method is an optimization technique originally devised by Nelder and Mead [29] and is based on geometric considerations. The geometric figure whose vertices are defined by a set of *n* +1 in an *n*-dimensional space is called simplex. For example, for two variables, a simplex is a triangle. The simplex algorithm compares function values at the three vertices of the triangle where the worst vertex (e.g., the largest value for minimization problem) is discarded and replaced by a new one. By iterative process, a new triangle is formed and the search continues until the function values at the vertices become smaller and smaller. The size of the triangle is thus reduced until the coordinates of the optimum point are found [30]. The advantage of this method is its simplicity which needs only objective function evaluations and does not require first and higher order derivatives such as the gradient-based methods [31]. Moreover, it is computationally compact and effective with good convergence properties for most problems with parameters to be optimized less than ten [32]. The method is highly applicable for nonlinear multi-input multioutput systems without obtaining transfer function or mathematical formulation and hence suitable for finding local minimum of an objective function defined by several variables.

In the present study, the aim is to minimize the weighted integral squared error (WISE) products of the DC bus voltage controlled by the G-VSC and the BES buck-boost converters. The considered performance index can be expressed in the following general form:

(12) $$\begin{array}{c} W\text{ISE}=W_{1}{\text{ISE}}_{1}+W_{2}{\text{ISE}}_{2}, \end{array}$$

where *W*_1_ and *W*_2_ are the weighting factors used to signify the relative importance of the design specifications and ISE_1_ and ISE_2  _ are the partial indices used to penalize the deviation of the DC bus voltage [31]. Based on the performance index in (12), the objective function to search for optimal parameters for the PI controllers (i.e., PI1, PI3, and PI7) responsible for DC bus voltage regulation is formulated. For example, the objective function for the case of BES-based regulation together with current-mode control scheme of G-VSC is described as follows:

(13) OF(xdc)=∫T0T1W1(Vdc−Vdc,ref)2dt+∫T1TFW2(Vdc−Vdc,ref)2dt,

where **x**_dc_ represents the control parameters for the PI3 and PI7 controller gains, *T*_0_ is the time at which the reference is changed within the entire length of the simulation time, *T*_*F*_, and *T*_1_ is suitably selected intermediate point that permits the assignment of different weighting factors, *W*_1_ and *W*_2_, to the initial and later portions of the response. The values of *W*_1_ and *W*_2_ are selected depending on the proportional and integral gains selected during initial trial and error tuning of the PI controllers, preferably *W*_1_ > *W*_2_ to ensure stability of the system.

## 6. Results And Discussion

The effectiveness of the proposed control strategies in comparison to the conventional method is investigated through simulation in PSCAD/EMTDC. Validation of the system component models is first given, and then performance comparison of the simulation results with and without use of BES as well as with optimized control scheme is discussed.

### 6.1. Model Validation

Validation of the models is made by comparing the steady state PV module *I*-*V* curves and the BES discharge voltage curves, respectively, with the corresponding manufacturer data.

#### 6.1.1. PV Model

The parameter extraction method for the PV model is based on [17], with key equations given in (1)–(2).  Table 1 provides the parameter values of the PV module used in the simulation of PV model in PSCAD/EMTDC.

**Figure 7:**
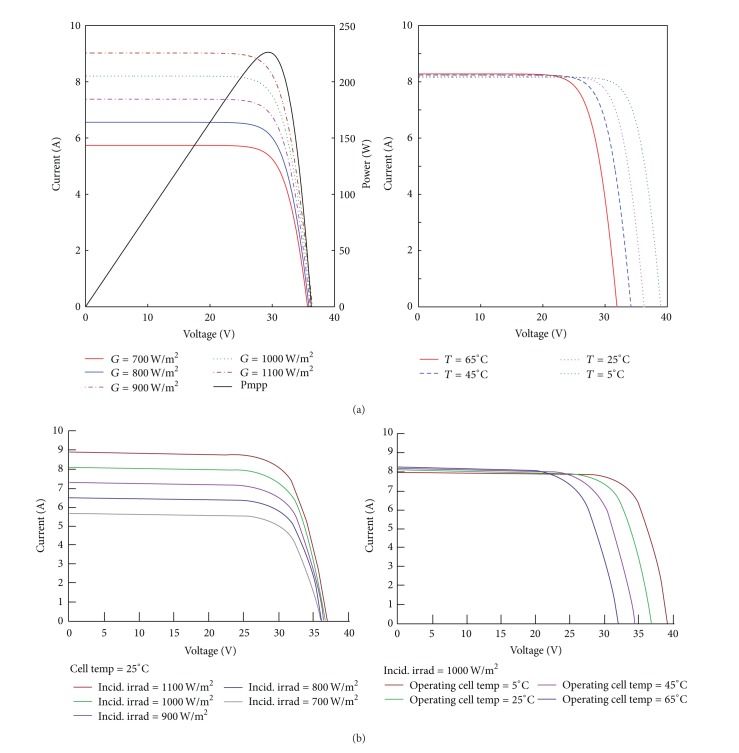
Comparison of the PV module *I*-*V* curves (a) simulated Hyundai M224SG and (b) manufacturer data.

The results from the simulated module's characteristic curves are compared with the Hyundai SG-series multicrystalline type solar module manufacturer datasheet [19].  Figure 7 shows the results of the comparison of the module's *I*-*V* characteristics simulated at standard test conditions (STCs). As can be seen in Figure 7, the simulated results (Figure 7(a)) are compared well to the values in the manufacturer datasheet (Figure 7(b)). The module's nominal power output is measured approximately by 226 W, as shown in Figure 7(a). It is evident that the behavior of the simulated PV module current increases with the increase in solar irradiation at constant temperature (25°C). Consequently, the voltage decreases with the increase in the cell's temperature, when the irradiation is fixed at 100 W/m^2^. Having validated the PV simulation model, the PV modules are arranged in series land parallel connections to form a PV array (generator). The PV generator size considered in the simulation is 215 kWp which is a typical size for grid-connected PV system found in residential areas.

#### 6.1.2. Battery Model

An NPC series valve regulated lead acid, namely, the Yuasa VRLA NP4-12cell (12 V, 4 Ah), is considered suitable for the cyclic application of the battery model [25]. Parameter values of the battery module are detailed in Table 2.

**Figure 8:**
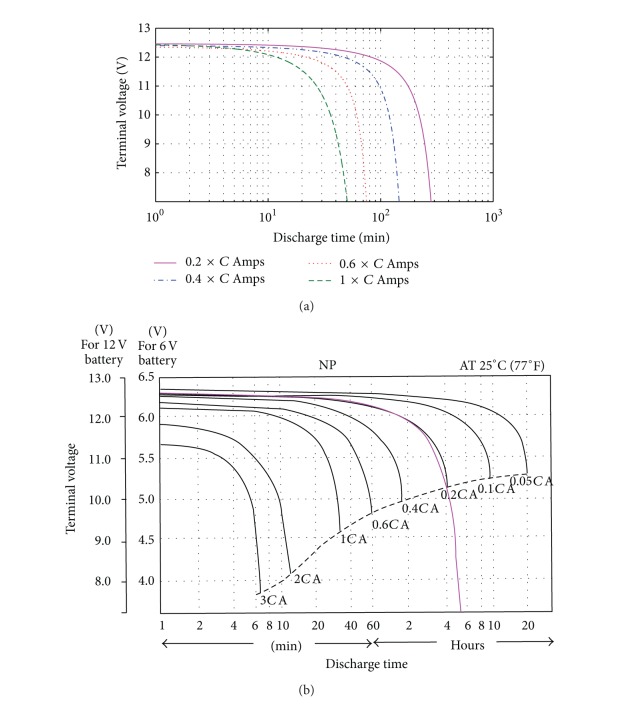
Comparison of the battery module discharge curves at different current magnitudes: (a) simulated Yuasa NP4-12 discharge curve and (b) manufacturer data of the Yuasa NP series battery (note that battery module's capacity *C = *C*_bat_ = 4 Ah). Note. *C*: given capacity as stated on each battery in Ah.

Figure 8 shows a comparison of the 12 V battery module simulation results with the battery data from the manufacturer in terms of the terminal voltage behaviors versus discharge time. The discharge currents ranging from 0.8 to 4 A have been simulated as shown in Figure 8(a). From the figure, all the discharge current curves of the simulated battery module are generally in good agreement with the standard manufacturer's discharge test data [25]. From Figure 8(b), by superimposing the voltage curve of 0.2 × *C*_bat_ ampere (0.8 A), the curve matches very well with the actual test curve during almost 80% of the discharge.

The BES simulation was developed by increasing *n*_*s*_ and *n*_*p*_ values, that is, the series and parallel arrangement of the battery module's matrix. In the simulation, a total of 48 kWh BES was used which can be arranged from approximately 20 series connected battery modules with 50 parallel strings. The BES output terminal voltage is around 240 V and it is interfaced with the DC/DC buck-boost converter that can step up the voltage to 500 V during boost mode. The battery bank is considered suitable for intermittent discharge to compensate for DC bus voltage discrepancies [28].

### 6.2. Simulation Results

The DC bus voltage regulation performance was evaluated by considering with and without the use of BES. The simulation parameters are as shown in Table 3.

#### 6.2.1. Evaluation of the Voltage-Mode Control Scheme of G-VSC

**Figure 9:**
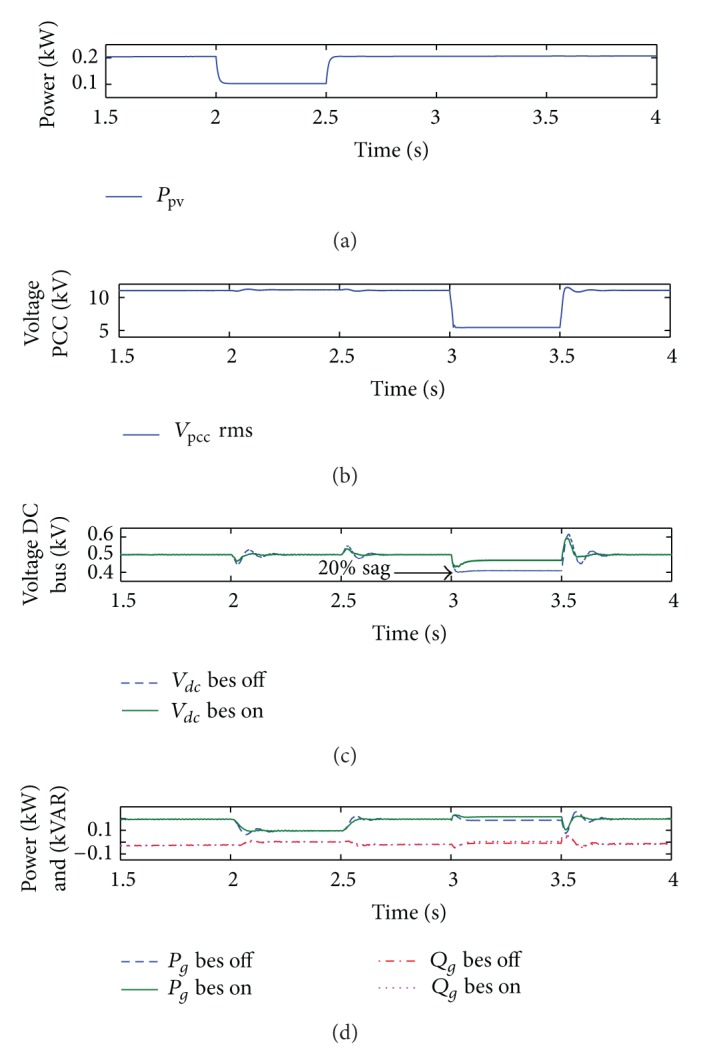
Results of DC bus voltage regulation and active and reactive power profiles for the case with and without BES and using voltage-mode controlled G-VSC.

Figure 9 shows the dynamic performance of the PV/BES system being subjected to critical a condition of ±50% step change of irradiation and a voltage sag at the PCC. As shown in Figure 9(a), an abrupt change in irradiation is applied at 2 s and lasted for 0.5 s which causes the array power to drop suddenly about 50%. Similarly, in Figure 9(b), when a voltage sag is applied at 3 s for a duration of 0.5 s, the PCC RMS voltage is found to be 5.5 kV, hence causing a 50% drop from the nominal 11 kV value.

Overall, as can be observed from the DC bus voltage profile of Figure 9(c), voltage fluctuation decreases when BES is connected as evident from the variation of transient peak surges and the corresponding transient settling times. Without BES, the transient spikes are measured around 10%–10.8% and 20%–23.2% (sag and spike), during irradiation disturbance and utility grid fault, respectively. However, when BES is connected, the spikes and sag are reduced to 6.6%–7.4% and 13.2%–18% (spikes with sag around 6.4%), respectively. These results imply that the BES compensation for transient overshoot and undershoot at the DC bus voltage results in minimal power fluctuation for the total power delivered to the utility grid (see Figure 9(d)).

Severe disturbance such as utility grid fault prevents the voltage-mode control method for the G-VSC to restore the DC bus voltage to its reference 500 V value, causing subsequent voltage sag for approximately 20% without BES. With BES connected, the voltage regulation is improved to a certain degree of approximately 6.4% sag because of the compensation through the BES converter. As shown in Figure 9(d), for the reactive power control, BES cannot overcome poor regulation of the voltage-mode control scheme, thus causing fluctuation of the reactive power during both disturbance conditions. This is due to the fact that BES compensation in this case only applies for active power regulation.

**Figure 10:**
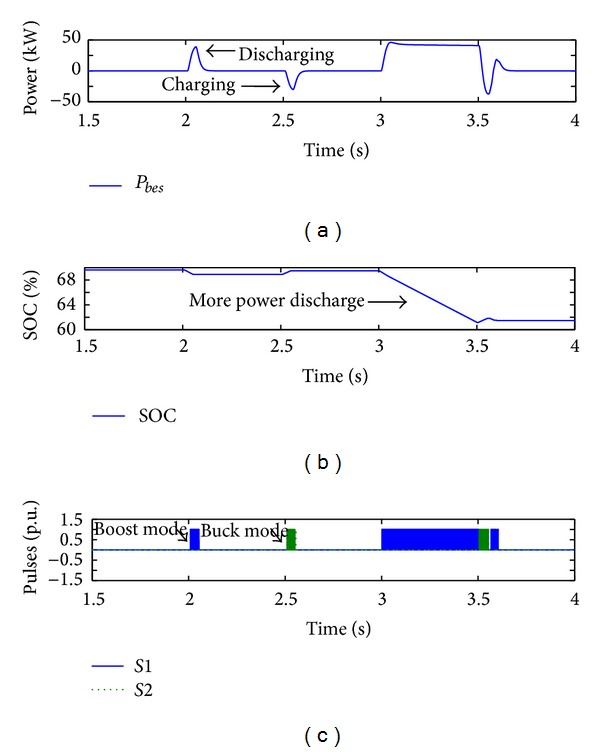
BES profiles during voltage regulation with voltage-mode control scheme of G-VSC.

During the simulation period of Figure 9, the BES output power and SOC profiles are recorded as shown in Figures 10(a) and 10(b), respectively. The switching states of the DC/DC buck-boost converter during regulation service are also shown in Figure 10(c).

As shown in Figures 10(a) and 10(b), BES discharged and charged its power to compensate for the DC bus voltage fluctuation, thus causing decrease/increase of SOC at 2 s and 2.5 s, respectively. However, more power is discharged during utility grid fault causing SOC to drop about 9% (see Figure 10(b)) in order to restore the resultant 20% drop of voltage of the DC bus in between 3 s and 3.5 s. It is evident that, although BES can slightly improve DC bus voltage regulation in the cases discussed, the voltage-mode control scheme of G-VSC shows poor performance and needs extra energy from the BES in order to enhance the DC bus voltage regulation.

#### 6.2.2. Evaluation of the Current-Mode Control Scheme of G-VSC

**Figure 11:**
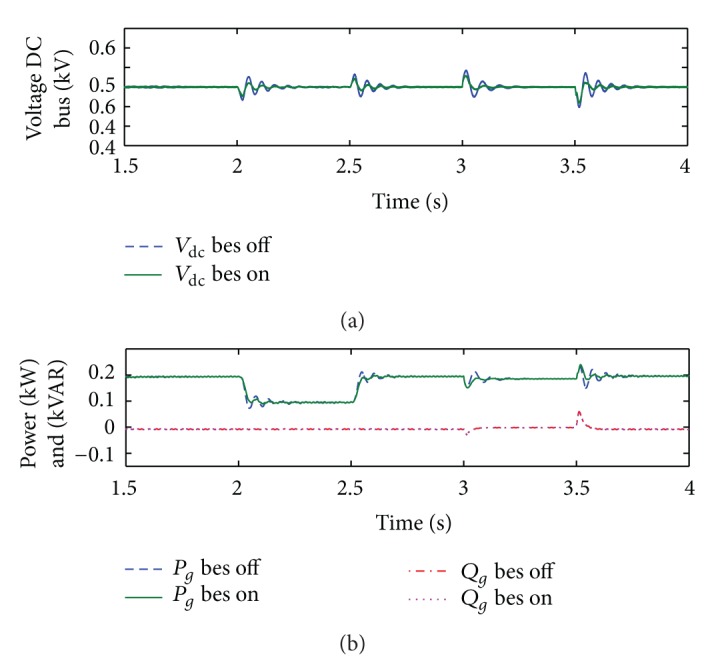
Results of DC bus voltage regulation and active and reactive power profiles for the case with and without BES and using current-mode controlled G-VSC.

The performance of PV/BES system using the current-mode control scheme of G-VSC is evaluated based on the similar disturbance conditions as previously discussed.  Figure 11 shows the overall system performance for the cases with and without BES.

From Figure 11, it is evident that the closed-loop control response of the current-mode scheme is relatively faster than that of the open-loop voltage-mode control scheme. The DC bus voltage profile as shown in Figure 11(a) is improved even without BES connected to the DC bus with 6.4%–6.8% maximum spikes during irradiation step change and 8.2%–10.2% range of spikes during utility grid fault. No voltage sag is observed during the latter disturbance condition. When BES is connected, the voltage profiles in terms of transient peaks and settling times (Figure 11(a)) are improved with only approximately 4.2%–4.8% and 5.8%–8.2% range of spikes during irradiation disturbance and utility grid fault, respectively. The results indicate the capability of BES in performing DC bus voltage regulation.

Overall comparison of the voltage-mode and current-mode control performances reveals that regulation by using the current-mode control scheme is superior, particularly when the system is subjected to utility grid faults. A more stable and fast response of system controller can be seen for the current-mode control method between 3 s and 3.5 s simulations as shown in Figures 11(a) and 11(b). It is important to note in Figure 11(b) that no change is observed for reactive power regulation with or without BES because BES is only set to compensate the bus voltage discrepancies using its active power, while G-VSC reactive power control works at unity power factor. However, it is obvious that reactive power regulation through current-mode control scheme of G-VSC is relatively better compared to the voltage-mode control scheme in controlling constant zero reactive power injection to the utility grid.

**Figure 12:**
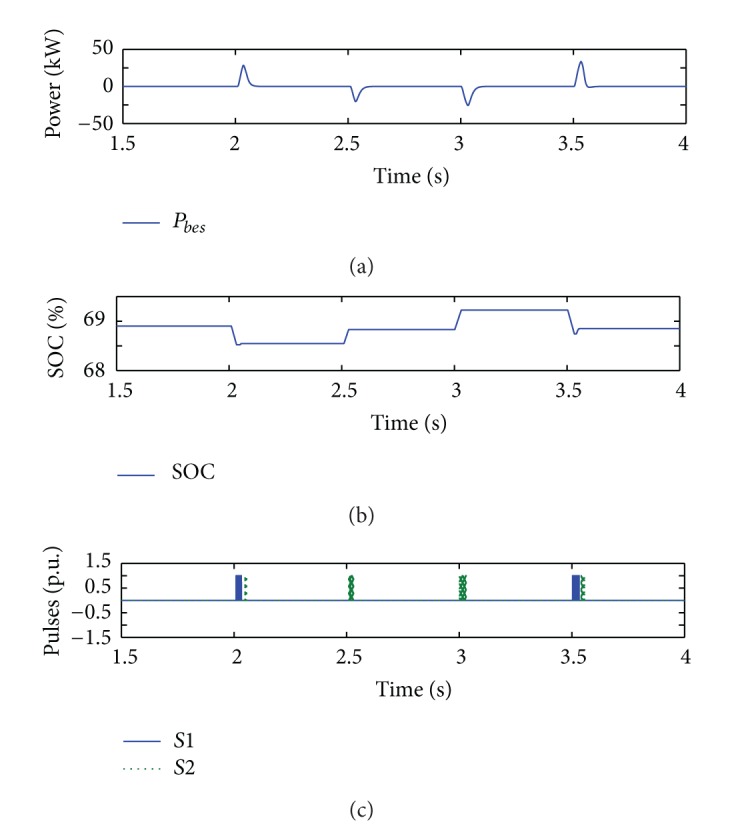
BES profiles during voltage regulation service of the system with current-mode control scheme of G-VSC.

Figure 12 shows the BES power and SOC profiles with the corresponding switching states of the DC/DC buck-boost converter. It is evident that improvement in system stability ensures optimal charging/discharging through the BES converter, in which minimum power from batteries is consumed/released during compensation, as shown in Figure 12(a). Small changes in SOC (Figure 12(b)) at the instant of disturbances applied indicate that the BES can be resized to a more optimal size which can be much smaller. Overall simulation results show that a combination of BES with current-mode control of G-VSC can provide superior DC bus voltage control performance of the grid-connected PV system.

#### 6.2.3. Results of DC Bus Voltage Regulation Using Simplex Optimized Control Schemes

**Figure 13:**
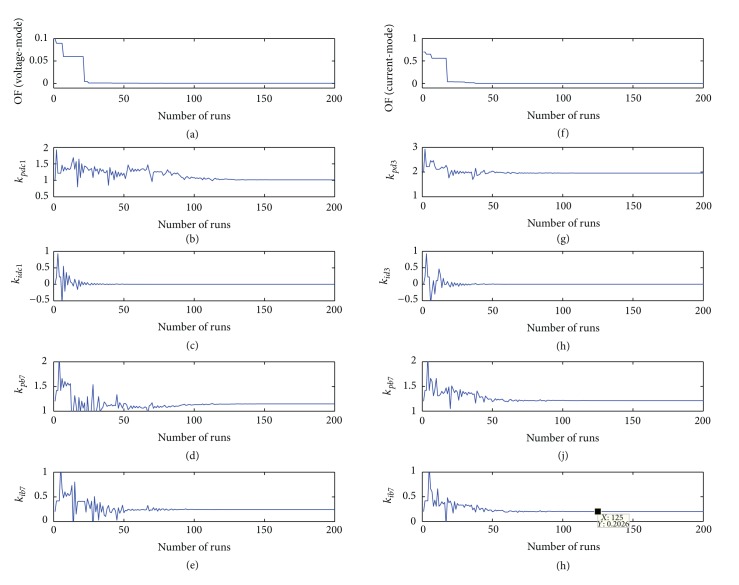
Optimization results of the BES control parameters and the parameters of voltage-mode and current-mode control schemes that are responsible for DC bus voltage regulation.

To compare the effectiveness of the proposed optimized control scheme with the traditional hand-tuning method of the power converters, several simulation cases were considered for the voltage- and current-mode controlled G-VSC with BES connected to the DC bus. The results of the parametric optimization problem using the voltage-mode and current-mode control schemes are as shown in Figure 13. From the figure, the objective function that is the WISE performance index of the DC bus voltage deviations has been reduced and the parameters become optimal after less than 100 numbers of iterations.

From the simulation results of Figure 13, a summary of the optimized control parameters used for the power converters described in Figure 1 is shown in Table 4.

**Figure 14:**
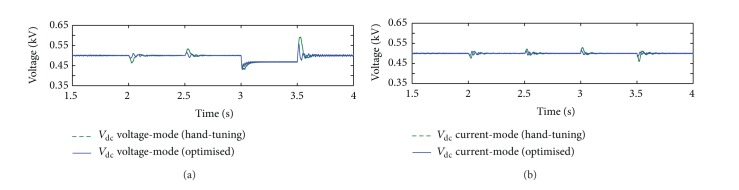
Comparison of DC bus voltage profiles for the cases without and with optimized control parameters: (a) voltage mode and (b) current mode.

Using the optimized controller gains shown in Table 4, simulations were carried out to compare the DC bus voltage regulation performance of the cases with BES using voltage-mode and current-mode controlled G-VSC.  Figure 14 shows the simulation results for the purpose of the comparison. In general, it is evident that, with optimized control schemes, the DC bus voltage can be further regulated at a certain level providing a more stable DC bus voltage over the period of varying disturbances conditions. As shown in Figure 14(a), when the system is subjected to changing atmospheric conditions, the optimized controller of the voltage-mode scheme can fairly improve voltage regulation. The undesired spikes are reduced from the range 6.6%–7.4% to 2.46%–2.7% at 2 s and 2.5 s, respectively. However, during voltage sag at the PCC, the DC bus voltage cannot be restored to its reference value. Again, this can be attributed to poor regulation of the voltage-mode control scheme which was based on the open-loop voltage control strategy [1]. Another reason is that BES operation is subjected to a maximum BES current discharge (e.g., at maximum of 1 × *C*_bes_ rate) in order to ensure safe operation so as to obey the current rating of the BES DC/DC converter.

However, with current-mode control scheme as shown in Figure 14(b), the optimized control parameters can significantly increase the regulation performance. The measured voltage spikes over the period of simulation are very marginal. From the figure, the voltage spikes have been reduced from the range 4.2%–4.8% to 1.82%–1.96% during irradiation disturbance and from 5.8%–8.2% to 2.12%–2.96% when voltage sag occurs at the PCC. This can significantly improve the performance of the G-VSC operation since the input voltage DC bus is now more stable and constant, thus contributing to minimizing loss of power within the power converter and a robust control system design.

## 7. Conclusion

This paper presented an assessment of the optimal control for DC bus voltage regulation by using a voltage-sourced converter (VSC) and a battery energy storage (BES) DC/DC buck-boost converter. The voltage-mode control method has a low number of control loops compared to the current-mode control scheme, making it simple in practice. However, the poor regulation performance of the voltage-mode control method results in greater energy requirement of batteries for compensation, which consequently increases storage cost. Using the BES compensation and voltage-mode controlled G-VSC, the DC bus voltage fluctuated at approximately 6.6% to 7.4% and 13.2% to 18% during the 50% step changes in the irradiation and utility grid faults (voltage sags), respectively. On the other hand, the overall performance was improved using the current mode control scheme, with fluctuations minimized to the range of 4.2% to 4.8% and 5.8% to 8.2% in the irradiation and utility grid faults (voltage sags), respectively. Moreover, the latter case causes no voltage sag to occur at the DC bus during the utility grid fault. These results can be attributed to the capability DC-coupled BES to restore DC bus voltage to 1 p.u. quickly. Furthermore, using the optimized current-mode control scheme, the DC bus voltage overshoot and undershoot only fluctuated in approximately 1.82% to 2.96% range during all disturbance cases. Overall, the results show that the proposed optimization method for PI control parameters can contribute to the optimal control of the power converters of the PV/BES system and improve the overall system efficiency.

## Figures and Tables

**Table 1:** PV module parameters.

Model parameter	Value	Symbols [17]
Cell type	6′′ multicrystalline silicon	—
Number of cells and connections	60 in series	—
Short circuit current at ^a^STC	8.3 A	*I*_sc,*r*_
Open circuit voltage at STC	36.7 V	*V*_oc,*r*_
Maximum power current at STC	7.7 A	*I*_mpp,*r*_
Maximum power voltage at STC	29.2 V	*V*_mpp,*r*_
Current temperature coefficient	0.004 A/K	*k*_*I*_
Voltage temperature coefficient	−0.1 V/K	*k*_*V*_
Diode ideality factor	1.5	*α*
Electron charge	1.602 × 10^−19^ C	*q*
Boltzmann's constant	1.380 × 10^−23^ J/K	*k*

^a^STC: standard test condition.

**Table 2:** Battery module parameters (12 V, 4 Ah).

Model parameter	Value	Symbols [21]
Cell type	Lead-acid (VRLA)	—
Number of cells per module	6 in series	—
Rated capacity	4 Ah	*Q*_rated_
Battery reserve	0.99 Ah	*Bat_resv *
Nominal capacity	0.85 Ah	*Q*_nom⁡_
Exponential capacity	0.67	*Q*_exp⁡_
Maximum voltage	12.15 V	*E*_full_
Exponential voltage	12.05 V	*E*_exp⁡_
Nominal voltage	12 V	*E*_nom⁡_
Charge current	4 A	*I*_chg_
Internal resistance	0.04	*R*_int⁡_
Efficiency	80%	*Eta *
^ a^Series battery	1	*n*_*s*_
^ a^Parallel battery	1	*n*_*p*_

^a^Increased combinations of *n*_*s*_ and *n*_*p*_ scales up the battery modules to form a BES.

**Table 3:** Simulation parameters.

Model parameter	Value	Symbols/comments
PV system	200 kWp	*P*_PV_
BES system	48 kWh	*P*_BES_
BES terminal voltage	240 V	*V*_bes_
BES total capacity	200 Ah	*C*_bes_
System loads	100 kW	*P*_*L*_
PCC system voltage	11 kV	*V*_pcc_ *LL*, *RMS*
G-VSC AC side voltage	0.23 kV	*V*_*t*_ *LL*, *RMS*
DC bus voltage	500 V	*V*_dc_
Capacitor	10,000 uF, 50,000 uF	*C*_PV_, *C*_dc_
Inductor	10 mH, 0.12 mH, 0.4 mH	*L*_1_, *L*_2_, *L*_*f*0_
Transformer (TR1)	0.1 MVA, 0.1 p.u.	Rating and leakage inductance
PI1, PI2 controller gains (G-VSC voltage mode)	1, 0.005, 0.2, 1	*k*_pdc1_, *k*_idc1_, *k*_pq2_, *k*_iq2_
PI3, PI4, PI5, PI6 controller gains (G-VSC current mode)	2, 0.001, 0.6, 0.05, 0.1, 20, 5, 0.05	*k*_pd3_, *k*_id3_, *k*_pq4_, *k*_iq4_, *k*_pd5_, *k*_id5_, *k*_pq6_, *k*_iq6_
PI7, PI8 controller gains (BES DC/DC converter)	1.2, 0.2, 1, 0.5	*k*_pb7_, *k*_ib7_, *k*_*p*8_, *k*_*i*8_
SOC limits	Max 0.95 p.u. Min 0.5 p.u.	^a^SOC_*H*_, SOC_*L*_
*V*_dc_ limits	Max 0.525 Min 0.475	^b^ *V*_dc,*H*_, *V*_dc,*L*_
Switching frequency	2500 Hz	DC/DC (MPPT/buck-boost) and G-VSC
Simulation time step and running duration	0.05 ms, 4 s	Δ*t*, *T*_*F*_

^a^Maximum 45% useable capacity, ^b^5% *V*_dc_ droop characteristic.

**Table 4:** Initial and optimised PI controller gains for the power converters based on simplex optimum run.

Power converter	Compensator	Hand tuning		^ a^Optimized	
		*k*_*p*_	*k*_*i*_	*k*_*p*_	*k*_*i*_
G-VSC (voltage mode)	PI1	1	0.005	**1.015**	**0.0008713**
	PI2	0.2	1	0.2	1

G-VSC (current mode)	PI3	2	0.001	**1.957**	**0.0002511**
	PI4	0.6	0.005	0.6	0.005
	PI5	0.1	20	0.1	20
	PI6	5	0.05	5	0.05

DC/DC buck-boost (BES)	PI7 (voltage-mode)	1.2	0.2	**1.147**	**0.2438**
	PI7 (current-mode)	1.2	0.2	**1.212**	**0.2026**
	PI8	1	0.5	1	0.5

^a^Minimize WISE performance index with *W*_1_ = 0.07, *W*_2_ = 0.008, total number of runs = 200.
